# Role of dorsal striatum circuits in relapse to opioid seeking after voluntary abstinence

**DOI:** 10.1038/s41386-024-01990-4

**Published:** 2024-09-19

**Authors:** Zilu Ma, Ying Duan, Ida Fredriksson, Pei-Jung Tsai, Ashley Batista, Hanbing Lu, Yavin Shaham, Yihong Yang

**Affiliations:** 1 Neuroimaging Research Branch, Intramural Research Program, National Institute on Drug Abuse, National Institute of Health, Baltimore, MD USA; 2 Center for Social and Affective Neuroscience, Linköping University, Linköping, Sweden; 3 Behavioral Neuroscience Branch, Intramural Research Program, National Institute on Drug Abuse, National Institute of Health, Baltimore, MD USA

**Keywords:** Addiction, Addiction

## Abstract

High relapse rate during abstinence is a defining characteristic of drug addiction. We previously found that opioid seeking progressively increases after voluntary abstinence induced by adverse consequences of oxycodone seeking (crossing an electric barrier). Functional MRI revealed that this effect is associated with changes in functional connectivity within medial orbitofrontal cortex (mOFC)- and dorsomedial striatum (DMS)-related circuits. Here, we used a pharmacological manipulation and fMRI to determine the causal role of mOFC and DMS in oxycodone seeking after electric barrier-induced abstinence. We trained rats to self-administer oxycodone (6 h/day, 14 days). Next, we induced voluntary abstinence by exposing them to an electric barrier for 2 weeks. We inactivated the mOFC and DMS with muscimol+baclofen (GABA_a_ and GABA_b_ receptor agonists) and then tested them for relapse to oxycodone seeking on abstinence days 1 or 15 without the electric barrier or oxycodone. Inactivation of DMS (*p* < 0.001) but not mOFC decreased oxycodone seeking before or after electric barrier-induced abstinence. Functional MRI data revealed that DMS inactivation decreased cerebral blood volume levels in DMS and several distant cortical and subcortical regions (corrected *p* < 0.05). Furthermore, functional connectivity of DMS with several frontal, sensorimotor, and auditory regions significantly increased after DMS inactivation (corrected *p* < 0.05). Finally, an exploratory analysis of an existing functional MRI dataset showed that DMS inactivation restored voluntary abstinence-induced longitudinal changes in DMS functional connectivity with these brain regions (*p* < 0.05). Results indicate a role of DMS and related brain circuits in oxycodone seeking after voluntary abstinence, suggesting potential targets for intervention.

## Introduction

A major obstacle in addressing the current opioid crisis is high relapse rates [1, 2]. In humans, re-exposure to drug-associated cues and contexts often triggers opioid relapse and craving [3]. In rodent models of relapse, drug seeking in the presence of cues and contexts associated with heroin or oxycodone self-administration progressively increases or incubates during homecage forced abstinence [4–7]. Recently, we found that incubation of oxycodone seeking also occurs after voluntary abstinence induced by introducing an electric barrier near the drug-paired lever [8]. We also found that in both males and females, incubation of opioid seeking is significantly stronger after electric barrier-induced than after forced abstinence.

In a subsequent study, we used a functional MRI (fMRI) protocol in rats [9, 10] to determine if longitudinal resting-state functional connectivity changes in orbitofrontal cortex (OFC) circuits predict incubation of opioid seeking after electric barrier-induced abstinence [11]. Previous studies have shown significant roles of lateral/ventral OFC in incubation of heroin and oxycodone seeking after forced abstinence [7, 12] and relapse to fentanyl seeking after food choice-induced abstinence [13]. We found that functional connectivity between the medial OFC (mOFC) and dorsomedial striatum (DMS), as well as DMS-related brain circuits, decreases from early to late voluntary abstinence [11]. Additionally, this reduction in functional connectivity is negatively correlated with the magnitude of the ‘incubation score’ (lever-presses on day 15 test minus lever-presses on day 1 test). However, the resting-state functional connectivity results are correlational. Therefore, the causal roles of mOFC and DMS and their related brain circuits in oxycodone seeking are unknown.

Here, we combined pharmacological reversible inactivation and fMRI to determine the causal role of mOFC and DMS in relapse to oxycodone seeking after electric-barrier induced abstinence. We first tested the effect of intracranial injections of the GABA_a_ and GABA_b_ receptor agonists muscimol and baclofen [14] into either mOFC or DMS on oxycodone relapse. We then used fMRI to determine the effect of DMS inactivation on changes in regional cerebral blood volume (CBV) and functional connectivity of DMS. The voxel-wise CBV reflects regional neuronal activity [15, 16], and voxel-wise functional connectivity of DMS reflects neuronal communications between DMS and other brain regions [17, 18]. Finally, we reanalyzed an existing fMRI dataset [11] to determine if DMS inactivation would restore decreased functional connectivity of DMS circuits due to electric barrier-induced abstinence. The goal of our fMRI part was to identify brain activity/connectivity patterns, including regional CBV and functional connectivity, due to DMS inactivation associated with the manipulation’s effect on oxycodone relapse.

## Methods and materials

### Subjects

Data were collected from 110 adult Sprague-Dawley rats (70 males and 40 females, postnatal 70–90 days, weighing 280–350 g and 200–250 g prior to surgery) obtained from ENVIGO (Indianapolis). Rats were maintained under a reverse 12-h:12-h light/dark cycle with food and water freely available. Experiments were conducted under protocols approved by the Animal Care and Use Committee of NIDA Intramural Research Program. 16 out of 110 rats were excluded due to catheter failure (*n* = 2), cannula misplacement (*n* = 9), poor health condition (*n* = 3), or poor image quality (*n* = 2). Both male and female rats were used in Experiment 1 (*n* = 78). Only male rats were used in the fMRI study in Experiment 2 (*n* = 14) and 3 (*n* = 18).

### Drugs

Oxycodone hydrochloride (HCl) was obtained from the NIDA pharmacy and diluted in sterile saline at a unit dose of 0.1 mg/kg for self-administration training [11, 19]. For Experiments 1 and 2, muscimol and baclofen (Sigma-Aldrich, M1523 and B5399) were dissolved in sterile saline and injected intracranially at a dose of 50 + 50 ng in 0.5 µl per side [13, 20, 21] 30 min before the relapse test or fMRI measurements.

### Overview of experiments

The details of the intravenous surgery, intracranial surgery, and the behavioral procedures of oxycodone self-administration, electric barrier-induced abstinence and relapse tests, the fMRI procedures and data analysis are provided in the Supplemental Online Material. Below we describe the procedures for Experiments 1–3.

#### Experiment 1: Effect of muscimol-baclofen inactivation of DMS or mOFC on oxycodone seeking after electric barrier-induced abstinence

**Fig. 1:**
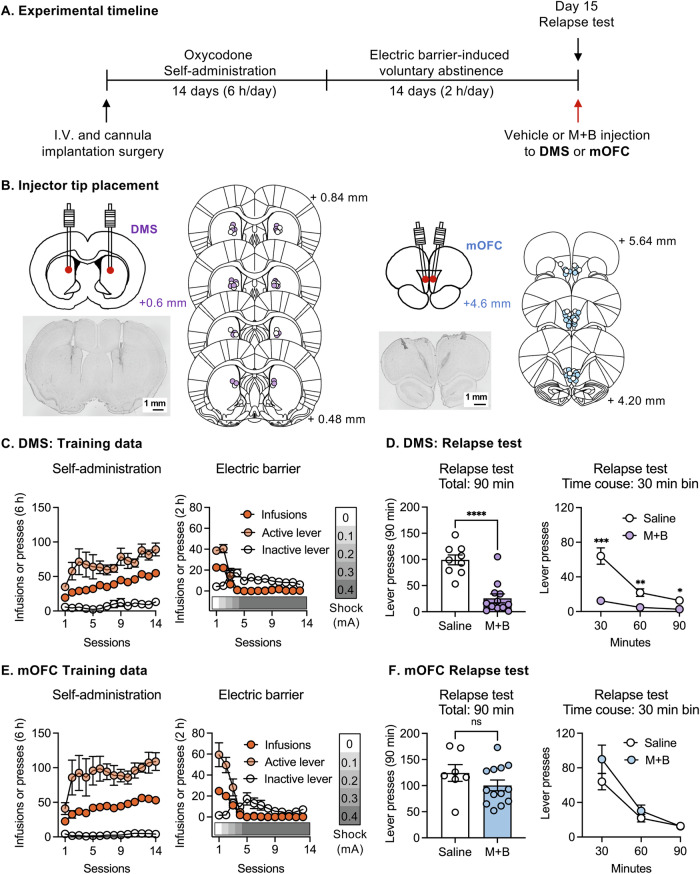
Effect of muscimol-baclofen (M + B) inactivation of DMS and medial OFC on oxycodone seeking after electric barrier-induced voluntary abstinence. **A** Experimental timeline. **B** Injector tip placement in the DMS or mOFC. **C**, **E** Oxycodone self-administration training (left) and electric barrier-induced voluntary abstinence (right) for DMS or mOFC rats. Data show mean ± SEM of number of infusions and active and inactive lever presses during the training phase. **D**, **F** Relapse test. Data show mean ± SEM number of active lever presses during the 90-min test sessions (left) or 30-min bin timecourse (right) after saline or muscimol-baclofen (50 + 50 ng per side) injection into **D** DMS or **F** medial OFC. ****p < 0.0001, ***p < 0.001, **p < 0.01, *p < 0.05. DMS: *n* = 9 to 12 rats per dose. mOFC: *n* = 6 to 13 rats per dose. DMS dorsomedial striatum. mOFC medial orbitofrontal cortex. M + B muscimol-baclofen.

As mentioned above, functional connectivity changes of mOFC and DMS circuits during electric barrier-induced abstinence predict ‘incubated’ oxycodone seeking [11]. The goal of Experiment 1 was to determine the causal role DMS or mOFC in oxycodone seeking after electric barrier-induced abstinence, using the classical muscimol-baclofen (M + B) inactivation procedure [14]. The experiment consisted of the following phases: oxycodone self-administration (14 days), electric barrier-induced abstinence (14 days), and day 15 oxycodone seeking test (Fig. 1A). On abstinence day 15, rats were tested for oxycodone seeking under extinction conditions after bilateral injection of Saline (0.5 µl per side) or M + B (50 + 50 ng in 0.5 µl per side) over 1 min through guide cannulas (26-gauge, Plastic One; 1.5 mm injector-projection from tip of cannulas) into the DMS (AP + 0.6, ML ± 2.1, DV − 4.2; *n* = 9 Saline, *n* = 12 M + B) or mOFC (AP + 4.65, ML ± 1.6, DV − 4, angle 16°; *n* = 7 Saline, *n* = 13 M + B) via a syringe pump (Harvard Apparatus). Injectors were left in place for 60 s to allow drug diffusion. Rats were placed in the testing chambers for 30 min and tested for relapse for 90 min.

We found that DMS but not mOFC M + B injections decreased oxycodone seeking on day 15 after electric barrier-induced abstinence. To determine the specificity of DMS inactivation on incubation, we trained and tested a separate cohort of rats for relapse to oxycodone seeking on day 1 after injections of vehicle or M + B into DMS (*n* = 11 Saline, *n* = 12 M + B). Furthermore, to ensure the decrease in oxycodonbe seeking was not due to motor or other performance deficits, we retrained the rats (*n* = 19 for day 15, *n* = 20 for day 1) to lever-press for palatable food pellets [22] and after 5 training days, we injected them with vehicle or M + B (counterbalanced order) prior to two test sessions separated by 2 days. At the end of the experiment, the rats were deeply anesthetized with isoflurane and their brains were extracted for cannula placement verification (Fig. 1B). See Supplemental Online Material for more details.

#### Experiment 2: effect of muscimol-baclofen inactivation of DMS on cerebral blood volume and functional connectivity

In Experiment 1, we found that M + B inactivation of DMS but not mOFC decreased relapse to oxycodone seeking after electric barrier induced voluntary abstinence. The goal of Experiment 2 was to explore the DMS-based brain circuits affected by the pharmacological inactivation using fMRI techniques. The experiment consisted of the following phases: oxycodone self-administration (14 days), electric barrier-induced voluntary abstinence (14 days), and day 15 fMRI experiment. On abstinence day 15, rats were scanned for resting-state fMRI and cerebral blood volumes (CBV)-based fMRI with pharmacological manipulations on DMS (*n* = 6 Saline, *n* = 6 M + B). All MRI data were acquired on a Bruker Biospin 9.4 T scanner (Bruker Medizintechnik). Saline or M + B at a dose of 50 + 50 ng in 0.5 µl/side was injected through bilateral MR-compatible guide cannulas into the DMS (AP + 0.6, ML ± 2.1, DV − 4.2; 1.5 mm injector-projection from tips of cannulas). Resting-state fMRI were collected both before (two 10-min scans) and 30 min after injection (two 10-min scans). CBV-based fMRI were collected using monocrystalline iron oxide nanoparticles (MION) Feraheme (AMAG Pharmaceuticals, ferumoxytol 510 mg/17 ml) as a contrast agent. One high-resolution structural image was collected for alignment and cannula placement verification. See Supplementary Online Materials for more detailed image acquisition (Fig. S1) and analysis procedure.

#### Experiment 3: effect of electric barrier-induced abstinence on longitudinal changes of DMS functional connectivity

In Experiment 2, we found that after electric barrier-induced abstinence, functional connectivity of DMS-based brain circuits was altered after pharmacological inactivation of the DMS. In Experiment 1, the DMS inactivation decreased relapse to oxycodone seeking after electric barrier-induced abstinence. The goal of Experiment 3 was to examine whether the decreased in functional connectivity of DMS-based circuits is related to incubated oxycodone seeking. For this purpose, we reanalyzed the resting-state fMRI data from our previous study [11]. The published experiment consisted of the following phases: oxycodone self-administration (14 days), electric barrier-induced voluntary abstinence (13 days), oxycodone seeking test and resting-state fMRI experiment (two 10-min scans) at both early (Day 1) and late (Day 15) abstinence phase. All MRI data were acquired on the same scanner as in Experiment 2. Please refer to Supplementary Online Materials for more detailed image acquisition and analysis procedure.

### Statistical analysis

Statistical results for voxelwise fMRI were corrected for multiple comparisons using the Monte Carlos simulation in AFNI (uncorrected *p* < 0.05, corrected *p* < 0.05, cluster size > 26 and > 33 for resting-state and CBV-based fMRI, respectively) as thresholds to determine significant brain voxels. Behavioral data and post-hoc fMRI data were analyzed with analysis of variances (ANOVAs), analysis of covariances (ANCOVAs), repeated-measures ANOVAs (RM-ANOVAs), RM-ANCOVAs (inactive lever as covariate), and two-sample t-test, using SPSS 29. For clarity, we only reported significant effects that are critical for data interpretation in Results and indicated the post-hoc analyses as asterisks in the figures. For comprehensive statistical report, see Supplementary Online Materials. In Experiment 1, there were no significant effects of sex or interactions between sex and the other independent variables. Thus, we combined the males and females for the data analyses and presentation.

## Results

### Experiment 1. Effect of inactivation of DMS or mOFC on oxycodone seeking after electric barrier-induced abstinence

We used a muscimol-baclofen (M + B) inactivation procedure to determine the causal roles of DMS and mOFC in oxycodone seeking after electric barrier-induced abstinence. All rats demonstrated reliable oxycodone self-administration, indicated by significant increases in both active lever-presses and number of infusions (Fig. 1C, E, left panels, Table S1). During the electric barrier phase, the rats voluntarily abstained from oxycodone taking when the shock intensity increased from 0 mA to 0.4 mA, indicated by significant decreases in number of infusions and active lever-presses (Fig. 1C, E, right panels).

On abstinence day 15, we injected saline or M + B into either the DMS or mOFC and tested for relapse to oxycodone seeking under extinction conditions. We found that M + B significantly decreased active lever-presses during the 90-min test session after DMS injections (ANCOVA F_1,18_ = 75.7, *p* < 0.001; Fig. 1D, Table S1). Additionally, the 30-min bin time course showed significant Group (Saline, M + B) × Time (30, 60, 90-min) interaction (two-way RM-ANCOVA F_2,32_ = 6.7, *p* = 0.004). M + B injections into the DMS had no effect on inactive lever-presses (Fig. S2), which were very low during the 90-min test (t-test_19_ = 0.07, *p* = 0.94). On a separate cohort of rats, we found that M + B injections into the DMS significantly decreased active lever-presses during the 90-min test session on abstinence day 1 (ANCOVA F_1,20_ = 14.4, *p* < 0.001; Fig. S3C).

In contrast, M + B injections into mOFC had no effect on oxycodone seeking after electric barrier-induced abstinence (Fig. 1F, Table S1). No significant group differences were found in active lever-presses during the 90-min test (ANCOVA F_1,17_ = 2.0, *p* = 0.17). The 30-min time course data showed significant effect of Time (two-way RM-ANCOVA F_2,30_ = 25.4, *p* < 0.001) but no significant effects of M + B dose or interaction between the two factors (*p* values > 0.1). M + B injections into the mOFC had no effect on inactive lever-presses, which were very low during the 90-min test (*t*-test_18_ = 0.12, *p* = 0.91).

Finally, inactivation of DMS with M + B had no significant effects on high-rate palatable food self-administration (Fig. S4), suggesting that the inhibitory effect of DMS inactivation on oxycodone seeking is not due to motor or other performance deficits.

### Experiment 2. Effect of DMS inactivation on cerebral blood volume and functional connectivity

In Experiment 1, we found that inactivation of DMS but not mOFC decreased relapse to oxycodone seeking after electric barrier-induced voluntary abstinence. In Experiment 2, we combined this rat model with fMRI to further explore changes in whole-brain CBV and in DMS-based functional connectivity induced by DMS inactivation.

**Fig. 2:**
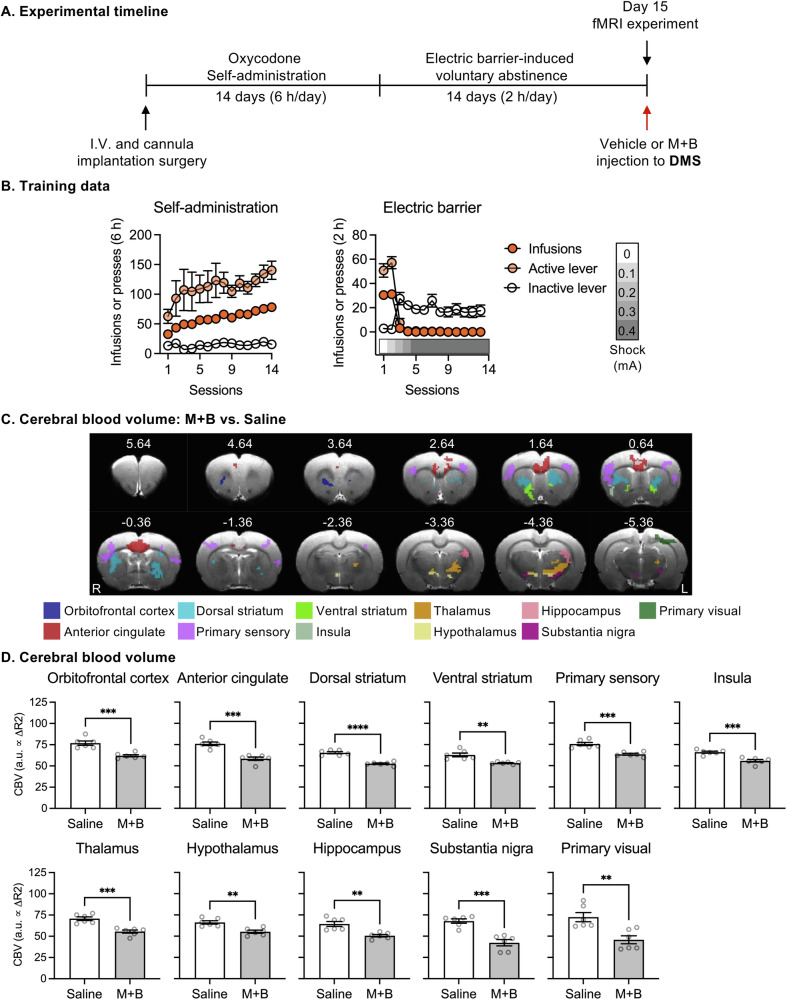
Effect of muscimol-baclofen (M + B) inactivation of DMS on brain cerebral blood volume. **A** Experimental timeline. **B** Oxycodone self-administration training (left) and electric barrier-induced voluntary abstinence (right). Data show mean ± SEM number of infusions and active and inactive lever presses during the training phase. **C** Voxelwise two-sample t-test of cerebral blood volume between rats injected with saline or muscimol-baclofen (50 + 50 ng per side) into the DMS. **D** Regional cerebral blood volume of clusters in **C**. ****p < 0.0001, ***p < 0.001, **p < 0.01, *n* = 6 rats per dose. M + B: muscimol-baclofen. DMS dorsomedial striatum.

All rats demonstrated reliable oxycodone self-administration, indicated by significant increases in both active lever-presses and number of infusions (Fig. 2B, left panel, Table S2). During the electric barrier phase, rats voluntarily abstained from oxycodone self-administration during electric barrier phase, indicated by significant decreases in number of infusions and active lever-presses (Fig. 2B, right panel). On abstinence day 15, the rats were scanned for resting-state fMRI at baseline (pre-injection), and for resting-state fMRI and CBV-based fMRI 30-min after DMS injections of saline or M + B.

CBV levels, an indicator of regional neuronal activity, were significantly lower both near the DMS injection site, as well as several distal regions (Fig. 2C, voxel-wise two-sample *t*-test, corrected *p* < 0.05, Table S2) in M + B-injected than in Saline-injected rats. Post-hoc analysis showed significant lower CBV levels (two-sample *t*-test) in the anterior cingulate cortex (ACC), orbital frontal cortex (OFC), dorsal striatum (DS), ventral striatum (VS), primary sensory cortex (S1), and insula (INS), thalamus (Th), hypothalamus (HypTh), hippocampus (Hp), substantia nigra (SN), and primary visual cortex (V1) in M + B injected rats (Fig. 2D).

**Fig. 3:**
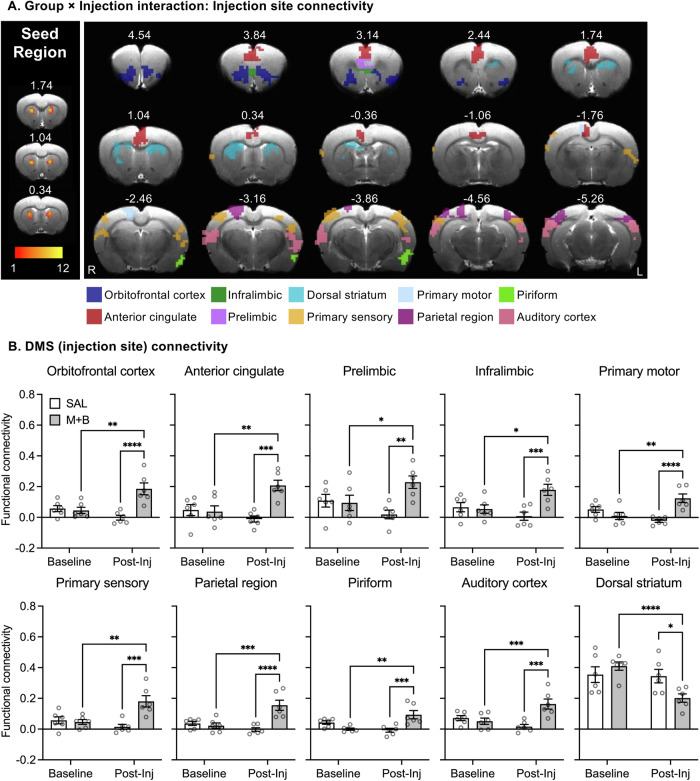
Effect of muscimol-baclofen (M + B) inactivation of DMS on brain functional connectivity. **A** Group by Injection interaction. (Left) Seed selection in the DMS based on injector tip placement for each rat. Seed size: 2 × 2 × 3 voxels near injector tip. Color scale indicate amount of seed overlap between rats. (Right) Group by Injection interaction after saline or muscimol-baclofen (50 + 50 ng per side) injections into DMS. **B** Functional connectivity between DMS and brain regions from **A** before and after the injections of saline or M + B. ****p < 0.0001, ***p < 0.001, **p < 0.01, *p < 0.05. DMS: *n* = 6 rats per dose. DMS: dorsomedial striatum. M + B: muscimol-baclofen. SAL saline.

We next assessed differential changes in DMS connectivity in the Saline and M + B groups before and after the injections. Two-way ANOVA revealed a significant Group (Saline, M + B) × Injection (Baseline, Post-injection) interaction in the DMS (injection site) connectivity with the OFC, ACC, infralimbic cortex (IL), prelimbic cortex (PrL), DS, S1, primary motor cortex (M1), parietal regions (PTL), piriform (Pir), and auditory area (Aud) (Fig. 3A, voxel-wise *F*-test, corrected *p* < 0.05, Table S2). Post-hoc analyses showed that the DMS (injection site) connectivity with DS significantly decreased after M + B injection (Fig. 3B). However, DMS connectivity with all other brain regions showed significant increases after M + B injection. In contrast, no changes in DMS connectivity were observed in Saline-injected group.

### Experiment 3. Effect of electric barrier-induced abstinence on longitudinal changes of DMS connectivity

**Fig. 4:**
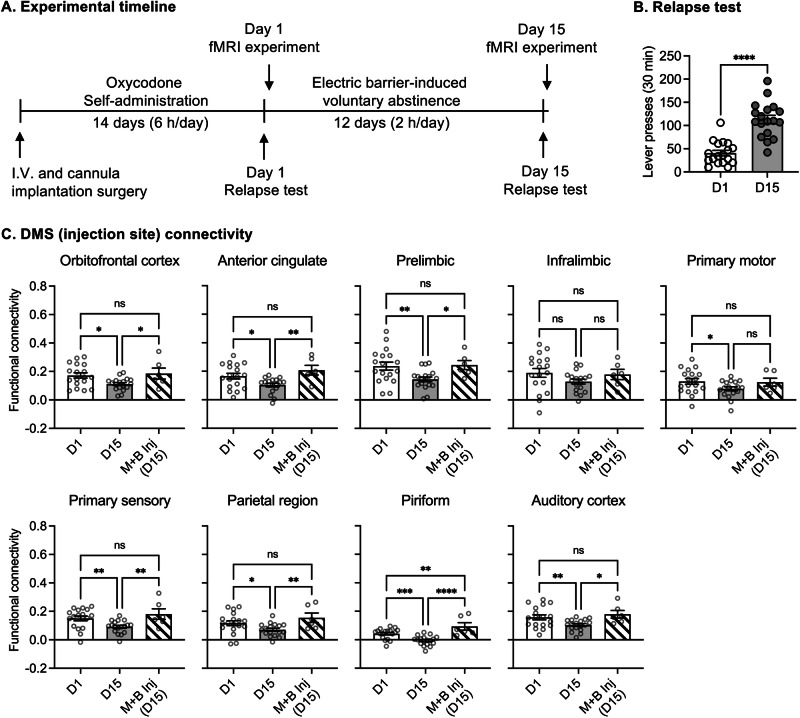
Effect DMS inactivation on functional connectivity of DMS-related circuits after electric barrier-induced abstinence (Experiment 2): comparison with functional connectivity of DMS-related circuits before and after electric barrier-induced abstinence from Ref. [11]. **A** Experimental timeline. **B** Relapse test. Data show means ± SEM number of active lever presses and individual data during the 30-min test sessions on abstinence days 1 and 15 before and after electric barrier-induced abstinence (within-subjects design). *n* = 18 rats (Data are from Ref. [11]). **C** Functional connectivity on day 1 and day 15 in comparison with functional connectivity in day 15 DMS inactivation in DMS-based brain circuits found in Experiment 2 (Fig. 3A). ****p < 0.0001, ***p < 0.001, **p < 0.01, *p < 0.05. D1: day 1. D15: day 15. M + B: muscimol-baclofen.

In Experiment 2, we found changes of functional connectivity in DMS-based brain circuits induced by pharmacological inactivation of the DMS, the same inactivation that decreased oxycodone seeking on Day 15 after electric barrier-induced abstinence in Experiment 1. Therefore, in Experiment 3, we used an existing dataset [11] with fMRI scans in early and late abstinence to examine whether functional connectivity in these DMS-based brain circuits is related to incubated oxycodone seeking (Fig. 4A). In that study (Ref. [11]), active lever-presses on abstinence day 15 after electric barrier-induced abstinence were significantly higher than on abstinence day 1 (Fig. 4B).

We next assessed functional connectivity of the DMS-based brain circuits on Day 1 and Day 15 using the DMS injection site and brain regions showing the Group × Injection interaction in Experiment 2 (Fig. 3A). Significant increases in functional connectivity of DMS with OFC, ACC, PrL, S1, Aud, PTL, and Pir were found in Day 15 compared to Day 1 (Fig. 4C, Table S3). Combining fMRI datasets from Experiments 2 and 3, our analyses indicate that DMS inactivation restored functional connectivity of these DMS circuits (except DMS-Pir) to the level of early abstinence (Day 1). Additionally, functional connectivity of DMS-Pir was “overcorrected” (higher than the level of Day 1).

## Discussion

We combined muscimol-baclofen pharmacological inactivation and fMRI to determine the role of mOFC and DMS and its related brain circuits in relapse to oxycodone seeking after electric barrier-induced abstinence, a rat model of relapse after voluntary abstinence induced by adverse consequences of opioid seeking [23, 24]. We found that inactivation of DMS but not mOFC decreased oxycodone seeking after electric barrier-induced abstinence. Our fMRI data showed that inactivation of DMS reduced neural activity levels at local injection site and in distant frontal regions, as indicated by reduced CBV levels. Additionally, functional connectivity of DMS with several frontal, sensorimotor, and auditory regions increased significantly after DMS inactivation. An exploratory analysis of our existing fMRI data [11] showed that inactivation of DMS restored the voluntary abstinence-induced longitudinal changes in functional connectivity of these brain circuits. Finally, DMS inactivation had no effect on high-rate operant responding for food pellets, indicating that decreased opioid seeking after DMS inactivation was not due to non-specific performance deficits. Together, our results indicate a causal role of DMS in relapse after electric barrier-induced abstinence and that this effect is associated with restoration of functional connectivity in DMS-related circuits.

### Inactivation of DMS but not medial OFC decreased oxycodone seeking after electric barrier-induced abstinence

We previously found that functional connectivity changes in mOFC- and DMS-related circuits during electric barrier-induced abstinence predict incubation of oxycodone seeking [11]. Here, we tested the causal role of these regions in oxycodone seeking after electric barrier-induced abstinence. We found that inactivation of DMS decreased oxycodone seeking after electric barrier-induced voluntary abstinence. This finding extends previous results on the role of dorsal striatum in drug relapse after forced or voluntary abstinence in rat models. Several studies reported that reversible inactivation or lesions of dorsolateral striatum (DLS) or local dopamine receptor blockade decrease cue-controlled cocaine and heroin seeking under a second-order schedule [25–27], cue-induced reinstatement of cocaine and heroin seeking after extinction [28, 29], relapse to cocaine and opioid (morphine) seeking after homecage forced abstinence seeking [29–31], and context-induced reinstatement of heroin seeking [32]. Other studies reported that blockade of dopamine receptors in DMS or DLS decreases incubation of methamphetamine seeking after forced abstinence [33, 34], and incubation of methamphetamine seeking after food choice-induced voluntary abstinence [20].

Together, our current results combined with these previous results suggest an important role of both DLS and DMS in cue-controlled drug seeking and drug relapse across drug classes and methods used to achieve abstinence: extinction, forced abstinence in homecage, food choice-induced abstinence, and electric barrier-induced abstinence. Of note, we also found that DMS inactivation decreased oxycodone seeking on abstinence day 1, before introducing the electric barrier. This suggests that the DMS plays a more general, abstinence period-independent, role in relapse to oxycodone seeking.

Unlike DMS inactivation, mOFC inactivation had no effect on oxycodone seeking after electric barrier-induced abstinence. Our negative results agree with a previous study showing that mOFC inactivation had no effect on extinction responding or cue-induced alcohol seeking [35]. In contrast, there is evidence for a role of lateral/ventral OFC in incubation of heroin and oxycodone seeking after homecage forced abstinence [7, 12], and relapse to fentanyl seeking after food choice-induced abstinence [13]. Additionally, reversible inactivation of lateral OFC decreases cue- and context-induced reinstatement of cocaine seeking after extinction [36, 37]. One possible explanation is that oxycodone relapse involves top-down control of motivated behavior from multiple cortical regions (e.g., lateral/ventral OFC, mPFC subregions). Thus, these other frontal cortical regions may compensate for the inactivation of the mOFC, leading to no observed change in relapse.

### Inactivation of DMS restores the longitudinal changes in functional connectivity of DMS-related circuits

Based on the results of Experiment 1, we used fMRI in combination with pharmacological inactivation to investigate the DMS-based brain circuitry that contributes to the effect of DMS inactivation on relapse to oxycodone seeking after electric barrier-induced abstinence. First, we found that inactivation of DMS significantly decreased neural activity, as indicated by lower CBV levels around the local injection site of DMS, as well as distant regions such as OFC, anterior cingulate cortex (ACC), insula, thalamus, substantia nigra, etc. Previous studies on DMS and its interconnected regions support this finding. A study on *Fos* expression shows that context-induced relapse to cocaine is associated with selective activation of DMS, DLS, ACC, and prefrontal cortex (PFC) [38]. Anatomical disconnection of the anterior intralaminar nuclei of thalamus to DMS projections decreases methamphetamine seeking after homecage abstinence [34]. Chemogenetic or optogenetic inhibition of DMS direct-pathway projection neurons at either their cell body or terminals in SN reticula (SNr), decreases reinstatement of cocaine [39] or alcohol [40] seeking. Optogenetic-induced long-term potentiation of the ventral/lateral OFC to DMS synapses reduces alcohol seeking [41]. Together, these findings suggest that the DMS, in conjunction with its interconnected regions, plays a significant role in relapse across drug classes and relapse-related procedures.

In addition to the reduction of local neural activity evidenced by altered CBV levels, inactivation of DMS also increased its functional connectivity with multiple frontal cortical regions. This suggests that while DMS inactivation may have decreased local synchrony, it also increased synchrony of DMS with distant cortical areas. This finding agrees with a study showing that chemogenetic inhibition of D1-expressing medium spiny neurons in the DMS increases its functional connectivity with ACC, prelimbic (PrL), and primary motor cortex [42]. Additionally, chemogenetic inhibition of mouse PFC increases functional connectivity with its direct thalamocortical targets [43]. Further in-vivo electrophysiology evidence suggest that this inhibition-induced increases in functional connectivity is associated with enhanced low-frequency (0.1–4 Hz) oscillatory power via suppression of high-frequency neural firing due to increased slow and δ band coherence [43].

Together, these studies support our finding that DMS inactivation decreases local functional connectivity but increases DMS functional connectivity with distant cortical regions. Future studies combining in-vivo electrophysiology and fMRI may provide further insights into changes in DMS-related functional connectivity changes and their role in relapse to oxycodone seeking.

Perhaps the most interesting finding in our study is that DMS inactivation ‘restored’ electric barrier-induced decreased or impaired DMS functional connectivity [11] with multiple brain regions. Indeed, the brain circuits showing increased functional connectivity due to the DMS inactivation in the current study are highly consistent with the DMS-related circuits showing decreased functional connectivity from early to late voluntary abstinence in our previous study [11]. This observation suggests that inactivation of DMS may have restored the normal function of DMS-based circuits that were impaired during electric barrier-induced abstinence.

To test this hypothesis, we used our existing fMRI data [11] and reanalyzed the functional connectivity between the muscimol-baclofen injection site (DMS) and brain areas showing inactivation-induced increases in functional connectivity in Experiment 2 (Fig. 3A). The functional connectivity between DMS and OFC, ACC, PrL, primary sensory cortex, parietal region, piriform, and auditory cortex was significantly decreased from early abstinence (day 1) to late abstinence (day 15) (Fig. 4C). Importantly, this decrease was restored when DMS was inactivated at the late abstinence day after electric barrier-induced abstinence.

These results suggest that inactivation of the DMS during the late abstinence decreased relapse to oxycodone seeking through restoration of multiple corticostriatal circuits that may have been impaired after oxycodone self-administration experience and subsequent electric barrier-induced abstinence.

Finally, the functional connectivity results in the present study and our previous fMRI study [11] may be relevant to human drug addiction. Previous studies showed that functional connectivity between the dorsolateral PFC (dlPFC) and caudate is decreased in people with heroin addiction [44]. Additionally, white matter track integrity between striatum and dlPFC predicts abstinence-induced changes in nicotine craving [45].

### Limitations

One methodological issue is the use of males only in the fMRI experiments. We made this choice because recent rodent studies suggest sex differences in brain anatomy and functional connectivity [46, 47]. Additionally, recent human studies suggest significant influences of ovarian cycle-dependent hormonal fluctuations on functional connectivity [48, 49]. These two factors would introduce significant variability and would require collection of fMRI data during estrus and non-estrus cycle, nearly tripling subject numbers. Furthermore, our fMRI procedure requires the use of light anesthesia (isoflurane and dexmedetomidine) to immobilize the rats, and rodent studies show both sex differences in and estrus cycle-dependent response to isoflurane [50] and dexmedetomidine [51]. This suggests that different concentrations of anesthetics are needed in female rats to achieve stable physiology, which confound data interpretation. Future studies using females are needed to determine the role of DMS-related functional connectivity and its role in opioid relapse across sexes.

Another methodological issue is that the rats were not exposed to relapse test prior to fMRI scan in Experiment 2. However, the rats in Experiment 1 experienced the same training (self-administration and voluntary abstinence) prior to the relapse test, as did the rats in Experiment 2. Therefore, the observed fMRI changes (M + B vs. saline injection) in Experiment 2 were expected to reflect the oxycodone seeking behavior as observed in the relapse test in Experiment 1. If a relapse test were performed prior to fMRI procedure, the changes in functional connectivity would reflect a combination of the training (self-administration and voluntary abstinence) experience and the relapse-test experience, which could confound data interpretation.

## Concluding remarks

In summary, our study showed a causal role of DMS in relapse to oxycodone seeking after electric barrier-induced voluntary abstinence. The changes in CBV levels and functional connectivity induced by M + B inactivation suggest that the manipulation restored the normal function of DMS-based mesocorticolimbic brain circuits that may have been impaired by oxycodone self-administration experience and subsequent electric barrier-induced abstinence. Further investigations on modulating DMS and DMS-related brain circuits using noninvasive neuromodulation approaches to mitigate opioid craving and relapse are warranted.

## Supplementary information

Supplementary Online Materials

## Data Availability

The behavioral and imaging data are available upon request (YY).
