# Supplementary material for: Role of dorsal striatum circuits in relapse to opioid seeking after voluntary abstinence
Source: Neuropsychopharmacology. 2024 Sep 19;50(2):452–60. doi: 10.1038/s41386-024-01990-4 (PMC11632082; doi:10.1038/s41386-024-01990-4)
Supplement: Supplementary file 1 — Supplementary Online Materials [file 41386_2024_1990_MOESM1_ESM.docx]

**Supplementary Online materials**

**Role of dorsal striatum circuits in relapse to opioid seeking after electric barrier-induced abstinence**

Zilu Ma, Ying Duan, Ida Fredriksson, Pei-Jung Tsai, Ashley Batista, Hanbing Lu,

Yavin Shaham, Yihong Yang

**Supplementary methods**

*Surgery*

In *Experiment 1 & 2*, rats were anesthetized with isoflurane (3 - 4 % induction, 1.5 ­– 2.5 % maintenance) during surgery. Customized intravenous catheters were made by attaching silastic catheters to a modified 22-gauge canula and fixed onto polypropylene mesh using dental cement. The customized catheter was then implanted into the jugular vein of the rats [1-3]. After the intravenous surgery, the rats were transferred to, and head fixed in the stereotaxic frame (Kopf Instruments) for intracranial implantation of cannula. Bilateral guide cannulas (26-gauge, Plastic One) were implanted 1.5 mm above either the DMS or mOFC using the following coordinates from bregma: AP +0.6, ML ±2.1, DV -4.2 for DMS; AP +4.65, ML ±1.6, DV -4, angle 16° for mOFC. Cannulas were then fixed on to the skull with dental cement (C&B Metabond) The rats were injected with ketoprofen (2.5 mg/kg) subcutaneously right after surgery and one day after surgery to decrease inflammation and relieve pain. The rats were given 7-10 days of recovery before behavior experiment trainings. The intravenous catheters were flushed with gentamicin (4.25 mg/ml) every 24 to 48 h during recovery and all experimental phases.

**Behavior experiments**

*Oxycodone self-administration*

After 7-10 days of recovery from surgery, the rats were trained on a fixed-ratio 1 (FR1) 20-s timeout schedule of reinforcement to self-administer oxycodone for 6 hours/day (six 1-h sessions separated by 10 min) for 14 days. The rats were trained in standard Med Associates self-administration chambers. Each session began with the illumination of a red houselight followed by the insertion of an active lever. Responses on the active lever resulted in one infusion of oxycodone (100 µl over 3.5 s at a unit dose of 0.1 mg/kg per infusion) paired with 20 s exposure to a compound tone-light cue; the white light cue was located above the active lever. During the 20-s tone-light exposure, responses on the active lever were not reinforced but recorded. Responses on the inactive lever had no consequences but were recorded. The maximum of infusions was limited to 15 per session. At the end of each session, active lever was retracted and houselight was turned off.

*Electric barrier-induced voluntary abstinence*

During this abstinence phase, oxycodone was available for 2-h/day. Parameters for the dose of oxycodone, reinforcement schedule, and tone-light cues were the same as in the self-administration training phase. The voluntary abstinence was achieved by introducing an electric barrier near the drug-paired active lever [4,5]. The “shock zone” (2/3 of the chamber near the drug-paired active lever) and “safe zone” (1/3 of the rest of the chamber) were separated by inserting a plastic block between the floor grids. The intensity of the current in the “shock zone” was set at 0.0 mA on the first day and was gradually increased to 0.3 mA at an increment of 0.1 mA per day. When rats approached the drug-paired active lever in the “shock zone”, they would receive a continuous mild footshock through the grid floor. The shock intensity was increased to 0.4 mA for rats that did not suppress oxycodone self-administration (< 3 reward/session). We did not increase beyond 0.4 mA if rats continue to press for oxycodone infusions.

*Relapse test*

In *Experiment 1 & 3*, we tested rats for relapse to oxycodone seeking under extinction conditions for 30 or 90 min during early (Day 1) and/or late (Day 15) abstinence. On relapse test day, the electric barrier was turned off and the plastic block was removed. All rats were habituated in the self-administration chamber for 30 min prior to start of the testing session to allow for exploration. Lever presses on the active lever resulted in the activation of infusion pump and delivery of oxycodone-paired tone-light cue, but not oxycodone infusions.

Food pellet self-administration and test

Before the first day of food self-administration training, rats were food restricted to homecage food (12 g/day) and maintained at this restriction level until the end of test phase. One the first day, rats were given 1 h magazine-training session which began with illumination of red houselight, followed by noncontingent delivery of 2 pellets per 2 min (TestDiet 45 mg pellet, 12.7% FAT, 66.7% carbohydrate, and 20.6% protein). Rats were then trained on an FR1 20-s timeout schedule of reinforcement to self-administer food pellets for 2 h/day (two 1-h sessions separated by 10 min) for 5 days. Each session began with the illumination of a red houselight followed by the insertion of an active lever. Responses on the active lever resulted dispensing of two pellets paired with 20 s exposure to a compound tone-light cue; the white light cue was located above the lever. During the 20-s tone-light exposure, responses on the active lever were not reinforced but recorded. Responses on the inactive lever had no consequences but were recorded. At the end of each session, active lever was retracted and houselight was turned off.

During the self-administration test, we injected vehicle or M+B to DMS (counterbalanced order, n = 19, within-subject) 30-min prior to two test sessions separated by 2 days. Saline (0.5 µl per side) or M+B (50 + 50 ng in 0.5 µl per side) were injected through bilateral guide cannulas (26-gauge, Plastic One) into the DMS via injectors (1.5 mm projection from tip of cannulas) using a syringe pump (Harvard Apparatus). Injectors were left in place for 60 s to allow for drug diffusion. Rats were placed in the testing chambers after the injection and 30 min later tested under same food self-administration condition for 2 h. Lever presses on the active lever resulted in dispensing of 2 pellets. Responses on the inactive lever had no consequences but were recorded.

**fMRI experiments**

*Animal preparation*

A combination of isoflurane and dexmedetomidine was used to anesthetize the rats during fMRI experiments [6,7]. The rats were initially anesthetized using 2.5% isoflurane in oxygen enriched air (70% N_2_ + 30% O_2_), followed by a bolus injection of dexmedetomidine (0.015 mg/kg, intraperitoneal). Then, the rats were transferred and head-fixed onto a customized MRI-compatible holder. Isoflurane was then gradually lowered and maintained at 0.5% - 0.75% through a nose cone. Continuous subcutaneous infusion of dexmedetomidine (0,015mg/kg/h) was delivered through an infusion pump (PHD 2000, Harvard Apparatus,). Multiple physiological parameters were monitored to ensure stable physiology during fMRI recording, which occurred at least 60 min after the introduction of anesthesia. Body temperature was monitored and maintained by a temperature-controlled water-heating pad during imaging. Heart rate, blood oxygenation level (MouseOx Pulse Oximeter, STARR Life Sciences Corp,) and respiration rate (Small animal Monitoring and Gating System, SA Instruments) were also monitored.

*Data acquisition and analysis for Experiment 2*

All MRI data were acquired on a Bruker Biospin 9.4T scanner (Bruker Medizintechnik, Karlsruhe, Germany) using a Birdcage coil for radiofrequency excitation and single-loop surface coil for recording. First, high-resolution T2-weighted structural image was collected using a Rapid Acquisition with Relaxation Enhancement sequence with the following parameters: repetition time (TR) = 3100 ms, echo time (TE) = 36 ms, field of view (FOV) = 30 × 30 cm^2^, in-plane matrix size = 256 × 256, slice thickness = 0.6 mm, slice gap = 0.1 mm, slice number = 31. Two 10-min baseline resting-state BOLD scans were collected using conventional gradient echo echo-planner imaging sequence with the following parameters: TR = 1500 ms, TE = 15 ms, FOV = 30 × 30 cm^2^, in-plane matrix size = 80 × 80, slice thickness = 0.6 mm, slice gap = 0.1 mm, slice number = 19. Then, vehicle (sterile saline) or muscimol and baclofen (50 +50 ng per side) were injected bilaterally into dorsal striatum (1.5 mm injector projection). After 30 minutes, three 10-min post-injection BOLD scans with same parameters were collected. For cerebral blood volume (CBV) MRI measurements, we used monocrystalline iron oxide nanoparticles (MION) Feraheme (AMAG Pharmaceuticals, ferumoxytol 510mg/17ml, Waltham, WA) as contrast agent. A pre-contrast multi-gradient echo (MGE) sequence was collected using the following parameters: TR = 600 ms, TE = 2 ms, FOV = 30 × 30, in-plane matrix size = 128 × 128, slice thickness = 0.765 mm, slice number = 17, echo number = 10, echo space = 2 ms. Feraheme was injected at a dose of 15 mg/kg through a tail vein catheter. A post-contrast MGE scan was then collected 2 minutes after the MION injection. See Figure S1 for schematic timeline of fMRI experiments.

All MGE data were preprocessed using an in-house MATLAB based processing pipeline [8]. Specifically, voxel-wise multi-echo MRI data was fitted to a single-exponential delay model:

$$S=S_{0}\times e^{(-TE \times R_{2}^{*})}$$

Here the $S_{0}$ represents MRI signal at TE = 0, $R_{2}^{*}$ represents the transverse-relaxation-rate. Since Feraheme is an intravascular contrast agent, changes in the $R_{2}^{*}$ pre- and post-MION injection (${\Delta R}_{2}^{*}$) is proportional to voxel-wise CBV [9]:

$$CBV \propto{\Delta R}_{2}^{*}$$

The ${\Delta R}_{2}^{*}$ was then normalized to signals from white matter to account for inter-animal variations [8]. We then carried out voxel-wise two-sample t-test between the MB and SAL groups to assess the differences in CBV levels after local injection into the DMS. All statistical results were corrected for multiple comparisons using the Monte Carlos simulation in AFNI (uncorrected p < 0.05, corrected p < 0.05 and cluster size > 33). We then extracted the CBV values from clusters of significant brain voxels for further analysis.

All BOLD fMRI data were preprocessed using Analysis of Functional NeuroImages (AFNI) [10], advanced normalization tools (ANTs, <http://stnava.github.io/ANTs/>) [11] and FMRIB Software Library (FSL, <https://fsl.fmrib.ox.ac.uk>) [12]. The pipeline includes distortion correction, skull removal, motion correction, co-registration of a standard template, noise removal using independent component analysis, band-pass filtering (0.01 - 0.1 Hz) and spatial smoothing (FWHM = 0.8 mm) [7,13]. We excluded two rats from further analysis due to excessive brain tissue damage near the cannula implantation site. We then conducted seed-based functional connectivity analysis for each BOLD fMRI scan. Seed regions selection in the dorsal striatum were based on 2 × 2 × 3 voxels near injector for each individual rats (Figure 3A). Seed-based functional connectivity maps for injector site were generated by calculating the whole-brain voxel-wise Pearson’s correlation coefficient with the time course of our selected dorsal striatum seed and then converted to z-scores. Subject-level connectivity maps for both baseline and post-injection were then generated by averaging the results from individual scans.

Next, we conducted a voxel-wise Group (SAL, MB) × Injection (BL, post-Inj) mixed-effect ANOVA to assess the differential effect of SAL and MB before and after injection. All statistical results were corrected for multiple comparisons using the Monte Carlos simulation in AFNI (uncorrected p < 0.05, corrected p < 0.05 and cluster size > 26) as thresholds to determine significant brain voxels. We then extracted the functional connectivity values from clusters of significant brain voxels for further analysis.

*Data acquisition and analysis for Experiment 3*

All MRI data were acquired on the same scanner with the same excitation and receiving coil as *Experiment 2*. First, high-resolution T2-weighted structural image was collected using a Rapid Acquisition with Relaxation Enhancement sequence with the following parameters: repetition time (TR) = 2200 ms, field of view (FOV) = 35 × 35 cm^2^, in-plane matrix size = 256 × 256, slice thickness = 1 mm, slice number = 23. Two 10-min baseline resting-state BOLD scans were collected using conventional gradient echo echo-planner imaging sequence with the following parameters: TR = 1000 ms, TE = 13 ms, FOV = 35 × 35 cm^2^, in-plane matrix size = 64 × 64, slice thickness = 1 mm, slice number = 17.

The data collected in *Experiment 3* were previously analyzed for the OFC connectivity in relationship with incubation of oxycodone seeking [7]. But in this study, we re-analyze the data for the DMS connectivity. All BOLD fMRI data were preprocessed using Analysis of Functional NeuroImages (AFNI) [10], advanced normalization tools (ANTs, <http://stnava.github.io/ANTs/>) [11] and FMRIB Software Library (FSL, <https://fsl.fmrib.ox.ac.uk>) [12]. The pipeline includes distortion correction, skull removal, motion correction, co-registration of a standard template, noise removal using independent component analysis, band-pass filtering (0.01 - 0.1 Hz) and spatial smoothing (FWHM = 0.6 mm). We then conducted seed-based functional connectivity analysis for each BOLD fMRI scan. Seed regions in the dorsal striatum were based on resampled seed used in *Experiment 2* (Figure 3A). Seed-based functional connectivity maps were generated by calculating the whole-brain voxel-wise Pearson’s correlation coefficient with the time course of our selected dorsal striatum seed and then converted to z-scores. Subject-level connectivity maps for both early and late abstinence were then generated by averaging the results from individual scans. We then extracted the functional connectivity values using clusters of significant brain voxels found in *Experiment 2* (Figure 3A) for further analysis.

**Supplementary tables**

**Table S1. Statistical analysis for Experiment 1.** Statistical analysis for the behavioral results (SPSS GLM repeated-measures module, GLM univariate module, and independent-samples T-test). Partial Eta^2^ ($\eta_{p}^{2}$) = proportion of explained variance. Cohen’s d ($d$) = standardized effect size. Observed power: computed using alpha = 0.05. RM: repeated measures; M+B: muscimol-baclofen.

| **Figure number** | **F- or T-value** | **p-value** | **Effect size** | **Observed power** |
| --- | --- | --- | --- | --- |
| Figure 1C. Self-administration infusions  RM-ANOVA  Within-subject factor: Session | Sessions (1-14) within-subject: F_13, 260_ = 24.64 | < 0.001* | $\eta_{p}^{2}$ = 0.55 | 1.00 |
| Figure 1C. Self-administration active lever presses  RM-ANOVA  Within-subject factors: Session, Lever | Sessions (1-14) within-subject: F_13, 260_ = 3.56  Lever (Active, inactive) within-subject: F_1, 20_ = 44.50  Lever × Session interaction: F_13, 260_ = 2.31 | < 0.001*  < 0.001*  0.006* | $\eta_{p}^{2}$ = 0.25  $\eta_{p}^{2}$ = 0.69  $\eta_{p}^{2}$ = 0.10 | 1.00  1.00  0.97 |
| Figure 1C. Electric barrier infusions  RM-ANOVA  Within-subject factor: Session | Sessions (1-13) within-subject: F_12, 240_ = 103.78 | < 0.001* | $\eta_{p}^{2}$ = 0.84 | 1.00 |
| Figure 1C. Electric barrier active lever presses  RM-ANOVA  Within-subject factors: Session, Lever | Sessions (1-13) within-subject: F_12, 240_ = 32.05  Lever (Active, inactive) within-subject: F_1, 20_ = 1.8  Lever × Session interaction: F_12, 240_ = 31.26 | < 0.001*  0.20  < 0.001* | $\eta_{p}^{2}$ = 0.62  $\eta_{p}^{2}$ = 0.082  $\eta_{p}^{2}$ = 0.61 | 1.00  1.00 |
| Figure 1D. Relapse test (total 90 min) active lever press  ANCOVA  Between-subject factor: Group  Covariate: inactive lever | Group (Saline, M+B) between-subject: F_1, 18_ = 75.69 | < 0.001* | $\eta_{p}^{2}$ = 0.81 | 1.00 |
| Figure 1D. Relapse test (30 min bin time course) active lever presses  RM-ANCOVA  between-subject factor: Group  within-subject factor: Time  Covariate: inactive lever (30 min bin) | Group (Saline, M+B) between-subject: F_1, 16_ = 19.30  Time (30, 60, 90) within-subject: F_2, 32_ = 15.22  Group × Time interaction: F_2, 32_ = 6.72 | < 0.001*  < 0.001*  0.004* | $\eta_{p}^{2}$ = 0.55  $\eta_{p}^{2}$ = 0.49  $\eta_{p}^{2}$ = 0.30 | 0.99  1.00  0.89 |
| Figure 1E. Self-administration infusions  RM-ANOVA  Within-subject factor: Session | Sessions (1-14) within-subject: F_13, 247_ = 9.88 | < 0.001* | $\eta_{p}^{2}$ = 0.34 | 1.00 |
| Figure 1E. Self-administration active lever presses  RM-ANOVA  Within-subject factors: Session, Lever | Sessions (1-14) within-subject: F_13, 247_ = 2.52  Lever (Active, inactive) within-subject: F_1, 19_ = 32.13  Lever × Session interaction: F_13, 247_ = 2.43 | 0.003*  <0.001*  0.004* | $\eta_{p}^{2}$ = 0.12  $\eta_{p}^{2}$ = 0.63  $\eta_{p}^{2}$ = 0.11 | 0.98  1.00  0.97 |
| Figure 1E. Electric barrier infusions  RM-ANOVA  Within-subject factor: Session | Sessions (1-13) within-subject: F_12, 228_ = 53.53 | < 0.001* | $\eta_{p}^{2}$ = 0.74 | 1.00 |
| Figure 1E. Electric barrier active lever presses  RM-ANOVA  Within-subject factors: Session, Lever | Sessions (1-13) within-subject: F_12, 228_ = 15.78  Lever (Active, inactive) within-subject: F_1, 19_ = 1.93  Lever × Session interaction: F_12, 228_ = 20.09 | < 0.001*  0.18  < 0.001* | $\eta_{p}^{2}$ = 0.45  $\eta_{p}^{2}$ = 0.092  $\eta_{p}^{2}$ = 0.51 | 1.00  1.00 |
| Figure 1F. Relapse test (total 90 min) active lever press  ANCOVA  Between-subject factor: Group  Covariate: inactive lever | Group (Saline, M+B) between-subject: F_1, 17_ = 2.04 | 0.17 | $\eta_{p}^{2}$ = 0.11 |  |
| Figure 1F. Relapse test (30 min bin time course) active lever presses  RM-ANCOVA  between-subject factor: Group  within-subject factor: Time  Covariate: inactive lever (30 min bin) | Group (Saline, M+B) between-subject: F_1, 15_ = 0.00  Time (30, 60, 90) within-subject: F_2, 30_ = 25.45  Group × Time interaction: F_2, 30_ = 0.55 | 0.99  < 0.001*  0.58 | $\eta_{p}^{2}$ = 0.00  $\eta_{p}^{2}$ = 0.63  $\eta_{p}^{2}$ = 0.035 | 1.00 |
| Supplementary Figure 2A. Relapse test (total 90 min) inactive lever press  Two-sample t-test | Saline vs. M+B: t = 0.073, df = 19 | 0.94 | $d$ = 0.032 |  |
| Supplementary Figure 2A. Relapse test (30 min bin time course) active lever presses  RM-ANOVA  between-subject factor: Group  within-subject factor: Time | Group (Saline, M+B) between-subject: F_1, 19_ = 0.12  Time (30, 60, 90) within-subject: F_2, 38_ = 0.94  Group × Time interaction: F_2, 38_ = 1,93 | 0.73  0.40  0.16 | $\eta_{p}^{2}$ = 0.006  $\eta_{p}^{2}$ = 0.047  $\eta_{p}^{2}$ = 0.092 |  |
| Supplementary Figure 2B. Relapse test (total 90 min) inactive lever press  Two-sample t-test | Saline vs. M+B: t = 0.12, df = 18 | 0.91 | $d$ = 0.054 |  |
| Supplementary Figure 2B. Relapse test (30 min bin time course) active lever presses  RM-ANOVA  between-subject factor: Group  within-subject factor: Time | Group (Saline, M+B) between-subject: F_1, 18_ = 0.048  Time (30, 60, 90) within-subject: F_2, 36_ = 7.40  Group × Time interaction: F_2, 36_ = 0.22 | 0.83  0.002*  0.81 | $\eta_{p}^{2}$ = 0.003  $\eta_{p}^{2}$ = 0.29  $\eta_{p}^{2}$ = 0.012 | 0.92 |
| Supplementary Figure 3B. Self-administration infusions  RM-ANOVA  Within-subject factor: Session | Sessions (1-14) within-subject: F_13, 286_ = 20.63 | < 0.001* | $\eta_{p}^{2}$ = 0.48 | 1.00 |
| Supplementary Figure 3B. Self-administration active lever presses  RM-ANOVA  Within-subject factors: Session, Lever | Sessions (1-14) within-subject: F_13, 286_ = 6.77  Lever (Active, inactive) within-subject: F_1, 22_ = 31.09  Lever × Session interaction: F_13, 286_ = 10.07 | < 0.001*  < 0.001*  < 0.001* | $\eta_{p}^{2}$ = 0.24  $\eta_{p}^{2}$ = 0.59  $\eta_{p}^{2}$ = 0.31 | 1.00  1.00  1.00 |
| Supplementary Figure 3C. Relapse test (total 90 min) active lever press  ANCOVA  Between-subject factor: Group  Covariate: inactive lever | Group (Saline, M+B) between-subject: F_1, 20_ = 14.44 | 0.001* | $\eta_{p}^{2}$ = 0.42 | 0.95 |
| Supplementary Figure 3C. Relapse test (30 min bin time course) active lever presses  RM-ANCOVA  between-subject factor: Group  within-subject factor: Time  Covariate: inactive lever (30 min bin) | Group (Saline, M+B) between-subject: F_1, 18_ = 16.59  Time (30, 60, 90) within-subject: F_2, 36_ = 28.35  Group × Time interaction: F_2, 38_ = 5.11 | < 0.001*  < 0.001*  0.01* | $\eta_{p}^{2}$ = 0.44  $\eta_{p}^{2}$ = 0.57  $\eta_{p}^{2}$ = 0.20 | 0.97  1.00  0.80 |
| Supplementary Figure 3D. Relapse test (total 90 min) Day 1 vs. Day 15 active lever press  ANCOVA  Between-subject factors: Group, Abstinence Day  Covariate: inactive lever day 1, inactive lever day 15 | Group (Saline, M+B) between-subject: F_1, 39_ = 87.07  Abstinence Day (D1, D15) between-subject: F_1, 39_ = 51.92  Group × Abstinence Day interaction: F_1, 39_ = 28.64 | < 0.001*  < 0.001*  < 0.001* | $\eta_{p}^{2}$ = 0.69  $\eta_{p}^{2}$ = 0.57  $\eta_{p}^{2}$ = 0.42 | 1.00  1.00  1.00 |
| Supplementary Figure 4B. Day 15 food self-administration test (30 min bin time course) pellet delivery  RM-ANOVA  Within-subject factor: Group, Time | Group (Saline, M+B) within-subject: F_1, 18_ = 1.54  Time (30, 60, 90, 120) within-subject: F_3, 54_ = 51.26  Group × Time interaction: F_3, 54_ = 1.87 | 0.23  < 0.001*  0.15 | $\eta_{p}^{2}$ = 0.079  $\eta_{p}^{2}$ = 0.74  $\eta_{p}^{2}$ = 0.094 | 1.00 |
| Supplementary Figure 4B. Day 15 food self-administration test (30 min bin time course) active lever presses  RM-ANOVA  Within-subject factor: Group, Time | Group (Saline, M+B) within-subject: F_1, 18_ = 0.50  Time (30, 60, 90, 120) within-subject: F_3, 54_ = 43.14  Group × Time interaction: F_3, 54_ = 0.35 | 0.49  < 0.001*  0.79 | $\eta_{p}^{2}$ = 0.027  $\eta_{p}^{2}$ = 0.71  $\eta_{p}^{2}$ = 0.019 | 1.00 |
| Supplementary Figure 4C. Day 1 food self-administration test (30 min bin time course) pellet delivery  RM-ANOVA  Within-subject factor: Group, Time | Group (Saline, M+B) within-subject: F_1, 20_ = 0.44  Time (30, 60, 90, 120) within-subject: F_3, 60_ = 29.39  Group × Time interaction: F_3, 60_ = 1.28 | 0.51  < 0.001*  0.29 | $\eta_{p}^{2}$ = 0.022  $\eta_{p}^{2}$ = 0.60  $\eta_{p}^{2}$ = 0.06 | 1.00 |
| Supplementary Figure 4C. Day 1 food self-administration test (30 min bin time course) active lever presses  RM-ANOVA  Within-subject factor: Group, Time | Group (Saline, M+B) within-subject: F_1, 20_ = 1.42  Time (30, 60, 90, 120) within-subject: F_3, 60_ = 34.12  Group × Time interaction: F_3, 60_ = 1.28 | 0.1  < 0.001*  0.29 | $\eta_{p}^{2}$ = 0.042  $\eta_{p}^{2}$ = 0.63  $\eta_{p}^{2}$ = 0.06 | 1.00 |

**Table S2. Statistical analysis for Experiment 2.** Statistical analysis for the behavioral results and fMRI results (SPSS GLM repeated-measures module and independent-samples T-test). Partial Eta^2^ ($\eta_{p}^{2}$) = proportion of explained variance. Cohen’s d ($d$) = standardized effect size. Observed power: computed using alpha = 0.05. RM: repeated measures; M+B: muscimol-baclofen; CBV: cerebral blood volume; FC: functional connectivity.

| **Figure number** | **F- or T-value** | **p-value** | **Effect size** | **Observed power** |
| --- | --- | --- | --- | --- |
| Figure 2B. Self-administration infusions  RM-ANOVA  Within-subject factor: Session | Sessions (1-14) within-subject: F_13, 143_ = 9.83 | < 0.001* | $\eta_{p}^{2}$ = 0.47 | 1.00 |
| Figure 2B. Self-administration active lever presses  RM-ANOVA  Within-subject factors: Session, Lever | Sessions (1-14) within-subject: F_13, 143_ = 2.14  Lever (Active, inactive) within-subject: F_1, 11_ = 36.20  Lever × Session interaction: F_13, 143_ = 1.80 | 0.015*  < 0.001*  0.05* | $\eta_{p}^{2}$ = 0.16  $\eta_{p}^{2}$ = 0.77  $\eta_{p}^{2}$ = 0.14 | 0.94  1.00  0.89 |
| Figure 2B. Electric barrier infusions  RM-ANOVA  Within-subject factor: Session | Sessions (1-13) within-subject: F_12, 132_ = 173.57 | < 0.001* | $\eta_{p}^{2}$ = 0.94 | 1.00 |
| Figure 2B. Electric barrier active lever presses  RM-ANOVA  Within-subject factors: Session, Lever | Sessions (1-13) within-subject: F_12, 132_ = 17.51  Lever (Active, inactive) within-subject: F_1,11_ = 8.97  Lever × Session interaction: F_12, 132_ = 45.82 | < 0.001*  0.012*  < 0.001* | $\eta_{p}^{2}$ = 0.61  $\eta_{p}^{2}$ = 0.45  $\eta_{p}^{2}$ = 0.81 | 1.00  0.78  1.00 |
| Figure 2C. CBV Orbitofrontal cortex  Two-sample t-test | Saline vs. M+B: t = 5.30, df = 10 | < 0.001* | $d$ = 3.06 | 1.00 |
| Figure 2C. CBV Anterior cingulate  Two-sample t-test | Saline vs. M+B: t = 6.21, df = 10 | < 0.001* | $d$ = 3.59 | 1.00 |
| Figure 2C. CBV Dorsal striatum  Two-sample t-test | Saline vs. M+B: t = 8.58, df = 10 | < 0.001* | $d$ = 4.96 | 1.00 |
| Figure 2C. CBV Ventral striatum  Two-sample t-test | Saline vs. M+B: t = 4.16, df = 10 | 0.002* | $d$ = 2.40 | 0.96 |
| Figure 2C. CBV Primary sensory  Two-sample t-test | Saline vs. M+B: t = 5.52, df = 10 | < 0.001* | $d$ = 3.19 | 1.00 |
| Figure 2C. CBV Insula  Two-sample t-test | Saline vs. M+B: t = 5.33, df = 10 | < 0.001* | $d$ = 3.08 | 1.00 |
| Figure 2C. CBV Thalamus  Two-sample t-test | Saline vs. M+B: t = 5.50, df = 10 | < 0.001* | $d$ = 3.17 | 1.00 |
| Figure 2C. CBV Hypothalamus  Two-sample t-test | Saline vs. M+B: t = 4.20, df = 10 | 0.002* | $d$ = 2.43 | 0.97 |
| Figure 2C. CBV Hippocampus  Two-sample t-test | Saline vs. M+B: t = 4.25, df = 10 | 0.002* | $d$ = 2.45 | 0.97 |
| Figure 2C. CBV Substantia nigra  Two-sample t-test | Saline vs. M+B: t = 5.67, df = 10 | < 0.001* | $d$ = 3.27 | 1.00 |
| Figure 2C. CBV Primary visual  Two-sample t-test | Saline vs. M+B: t = 3.73, df = 10 | 0.004* | $d$ = 2.15 | 0.92 |
| Figure 3B. FC Orbitofrontal cortex  RM-ANOVA  between-subject: Group  within-subject factor: Injection | Group (Saline, M+B) between-subject: F_1, 10_ = 26.15  Injection (BL, Post-Inj) within-subject: F_1, 10_ = 1.69  Group × Injection interaction: F_1, 10_ = 10.42 | < 0.001*  0.22  0.009* | $\eta_{p}^{2}$ = 0.72  $\eta_{p}^{2}$ = 0.15  $\eta_{p}^{2}$ = 0.51 | 1.00  0.83 |
| Figure 3B. FC Anterior cingulate  RM-ANOVA  between-subject: Group  within-subject factor: Injection | Group (Saline, M+B) between-subject: F_1, 10_ = 14.87  Injection (BL, Post-Inj) within-subject: F_1, 10_ = 2.27  Group × Injection interaction: F_1, 10_ = 9.55 | 0.003*  0.16  0.011* | $\eta_{p}^{2}$ = 0.60  $\eta_{p}^{2}$ = 0.19  $\eta_{p}^{2}$ = 0.49 | 0.93  0.85 |
| Figure 3B. FC Prelimbic  RM-ANOVA  between-subject: Group  within-subject factor: Injection | Group (Saline, M+B) between-subject: F_1, 10_ = 5.29  Injection (BL, Post-Inj) within-subject: F_1, 10_ = 0.33  Group × Injection interaction: F_1, 10_ = 8.34 | 0.044*  0.58  0.016* | $\eta_{p}^{2}$ = 0.35  $\eta_{p}^{2}$ = 0.032  $\eta_{p}^{2}$ =0.46 | 0.53  0.84 |
| Figure 3B. FC Infralimbic  RM-ANOVA  between-subject: Group  within-subject factor: Injection | Group (Saline, M+B) between-subject: F1, 10 = 7.15  Injection (BL, Post-Inj) within-subject: F1, 10 = 1.19  Group × Injection interaction: F1, 10 = 8.94 | 0.023*  0.30  0.014* | $\eta_{p}^{2}$ = 0.42  $\eta_{p}^{2}$ = 0.11  $\eta_{p}^{2}$ = 0.47 | 0.67  0.87 |
| Figure 3B. FC Primary motor  RM-ANOVA  between-subject: Group  within-subject factor: Injection | Group (Saline, M+B) between-subject: F_1, 10_ = 5.91  Injection (BL, Post-Inj) within-subject: F_1, 10_ = 1.04  Group × Injection interaction: F_1, 10_ = 21.21 | 0.035*  0.33  < 0.001* | $\eta_{p}^{2}$ = 0.37  $\eta_{p}^{2}$ = 0.094  $\eta_{p}^{2}$ = 0.68 | 0.60  0.98 |
| Figure 3B. FC Primary sensory  RM-ANOVA  between-subject: Group  within-subject factor: Injection | Group (Saline, M+B) between-subject: F_1, 10_ = 10.62  Injection (BL, Post-Inj) within-subject: F_1, 10_ = 3.28  Group × Injection interaction: F_1, 10_ = 12.15 | 0.009*  0.10  0.006* | $\eta_{p}^{2}$ = 0.52  $\eta_{p}^{2}$ = 0.25  $\eta_{p}^{2}$ = 0.55 | 0.83  0.88 |
| Figure 3B. FC Parietal region  RM-ANOVA  between-subject: Group  within-subject factor: Injection | Group (Saline, M+B) between-subject: F_1, 10_ = 11.14  Injection (BL, Post-Inj) within-subject: F_1, 10_ = 5.49  Group × Injection interaction: F_1, 10_ = 18.69 | 0.008*  0.041*  0.002* | $\eta_{p}^{2}$ = 0.53  $\eta_{p}^{2}$ = 0.35  $\eta_{p}^{2}$ = 0.65 | 0.85  0.56  0.97 |
| Figure 3B. FC Piriform  RM-ANOVA  between-subject: Group  within-subject factor: Injection | Group (Saline, M+B) between-subject: F_1, 10_ = 4.44  Injection (BL, Post-Inj) within-subject: F_1, 10_ = 1.47  Group × Injection interaction: F_1, 10_ = 15.65 | 0.061  0.25  0.003* | $\eta_{p}^{2}$ = 0.31  $\eta_{p}^{2}$ = 0.13  $\eta_{p}^{2}$ = 0.61 | 0.95 |
| Figure 3B. FC Auditory cortex  RM-ANOVA  between-subject: Group  within-subject factor: Injection | Group (Saline, M+B) between-subject: F_1, 10_ = 5.09  Injection (BL, Post-Inj) within-subject: F_1, 10_ = 4.20  Group × Injection interaction: F_1, 10_ = 37.05 | 0.048*  0.068  < 0.001* | $\eta_{p}^{2}$ = 0.34  $\eta_{p}^{2}$ = 0.30  $\eta_{p}^{2}$ = 0.79 | 0.53  1.00 |
| Figure 3B. FC Dorsal striatum  RM-ANOVA  between-subject: Group  within-subject factor: Injection | Group (Saline, M+B) between-subject: F_1, 10_ = 0.70  Injection (BL, Post-Inj) within-subject: F_1, 10_ = 43.08  Group × Injection interaction: F_1, 10_ = 35.56 | 0.42  < 0.001*  < 0.001* | $\eta_{p}^{2}$ = 0.065  $\eta_{p}^{2}$ = 0.81  $\eta_{p}^{2}$ = 0.78 | 1.00  1.00 |

**Table S3. Statistical analysis for Experiment 3.** Statistical analysis for the behavioral results and fMRI results (SPSS GLM repeated-measures module and GLM univariate module). Partial Eta^2^ ($\eta_{p}^{2}$) = proportion of explained variance. Observed power: computed using alpha = 0.05. RM: repeated measures; M+B: muscimol-baclofen; D1: Day 1; D15: Day 15

| **Figure number** | **F- or T-value** | **p-value** | **Effect size** | **Observed power** |
| --- | --- | --- | --- | --- |
| Figure 4B. Relapse test (total 30 min) active lever press  RM-ANOVA  Within-group factor: Abstinence Day  Covariate: inactive lever day 1, inactive lever day 15 | Abstinence Day (D1, D15) within-subject: F1, 33 = 44.00 | < 0.001* | $\eta_{p}^{2}$ = 0.57 | 1.00 |
| Figure 4C. FC Orbitofrontal cortex  ANOVA  between-subject: Group | Group (D1, D15, D15 M+B) between-subject: F_2, 39_ = 4.58 | 0.016* | $\eta_{p}^{2}$ = 0.19 | 0.74 |
| Figure 4C. FC Anterior cingulate  ANOVA  between-subject: Group | Group (D1, D15, D15 M+B) between-subject: F_2, 39_ = 5.93 | 0.006* | $\eta_{p}^{2}$ = 0.23 | 0.85 |
| Figure 4C. FC Prelimbic  ANOVA  between-subject: Group | Group (D1, D15, D15 M+B) between-subject: F_2, 39_ = 5.05 | 0.011* | $\eta_{p}^{2}$ = 0.21 | 0.79 |
| Figure 4C. FC Infralimbic  ANOVA  between-subject: Group | Group (D1, D15, D15 M+B) between-subject: F_2, 39_ = 1.62 | 0.21 | $\eta_{p}^{2}$ = 0.076 |  |
| Figure 4C. FC Primary motor  ANOVA  between-subject: Group | Group (D1, D15, D15 M+B) between-subject: F_2, 39_ = 2.55 | 0.091 | $\eta_{p}^{2}$ = 0.12 |  |
| Figure 4C. FC Primary sensory  ANOVA  between-subject: Group | Group (D1, D15, D15 M+B) between-subject: F_2, 39_ = 6.18 | 0.005* | $\eta_{p}^{2}$ = 0.24 | 0.87 |
| Figure 4C. FC Parietal region  ANOVA  between-subject: Group | Group (D1, D15, D15 M+B) between-subject: F_2, 39_ = 4.66 | 0.015* | $\eta_{p}^{2}$ = 0.19 | 0.75 |
| Figure 4C. FC Piriform  ANOVA  between-subject: Group | Group (D1, D15, D15 M+B) between-subject: F_2, 39_ = 15.36 | < 0.001* | $\eta_{p}^{2}$ = 0.44 | 1.00 |
| Figure 4C. FC Auditory cortex  ANOVA  between-subject: Group | Group (D1, D15, D15 M+B) between-subject: F_2, 39_ = 5.40 | 0.009* | $\eta_{p}^{2}$ = 0.22 | 0.82 |

**Supplementary figures**

**
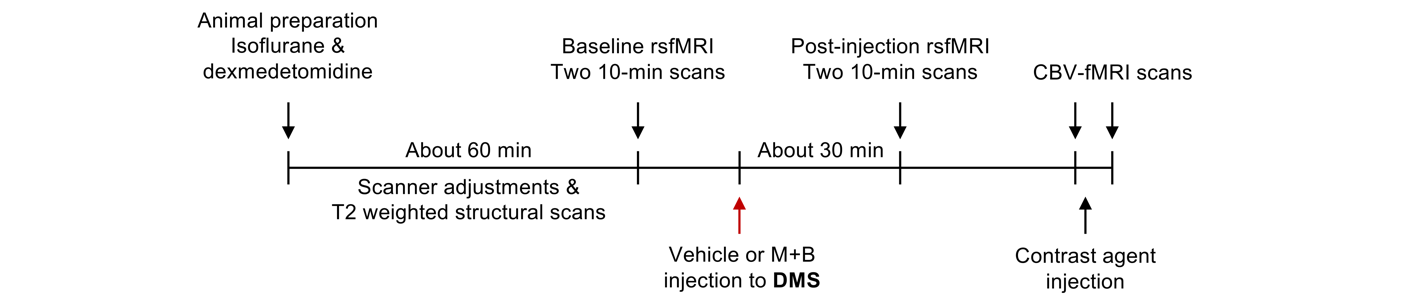
**

**Supplementary Figure 1: Experimental timeline of fMRI scans.** rsfMRI: resting-state functional MRI. DMS: dorsomedial striatum. M+B: muscimol-baclofen. CBV: cerebral blood volume.

**
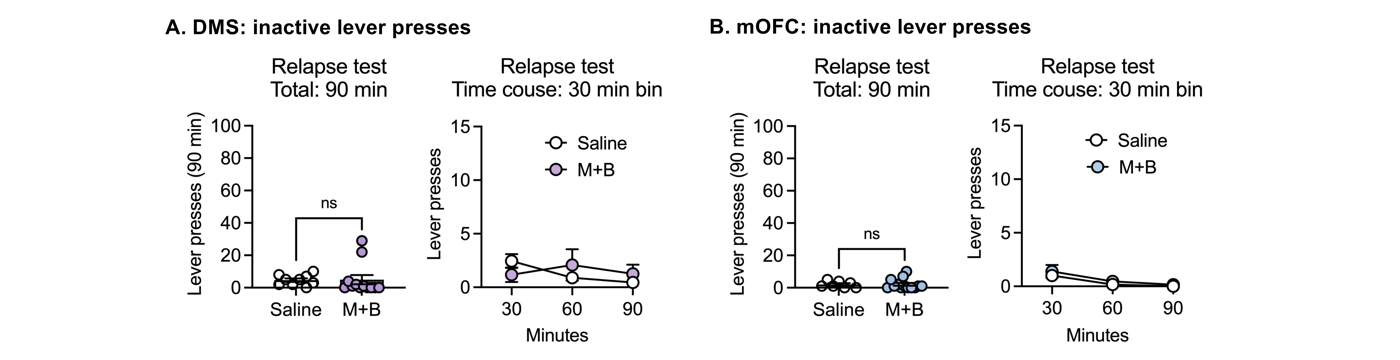
**

**Supplementary Figure 2: Effect of muscimol-baclofen (M+B) inactivation of DMS or mOFC on inactive lever presses of oxycodone seeking after electric barrier-induced voluntary abstinence.** Data show mean ± SEM number of inactive lever presses during the 90-min test sessions (left) or 30-min bin timecourse (right) after saline or muscimol-baclofen (50 + 50 ng per side) injection into (**A**) DMS. (**B**) mOFC. DMS: n = 9 to 12 rats per dose. mOFC: n = 6 to 13 rats per dose. DMS: dorsomedial striatum. mOFC: medial orbitofrontal cortex. M+B: muscimol-baclofen.

**
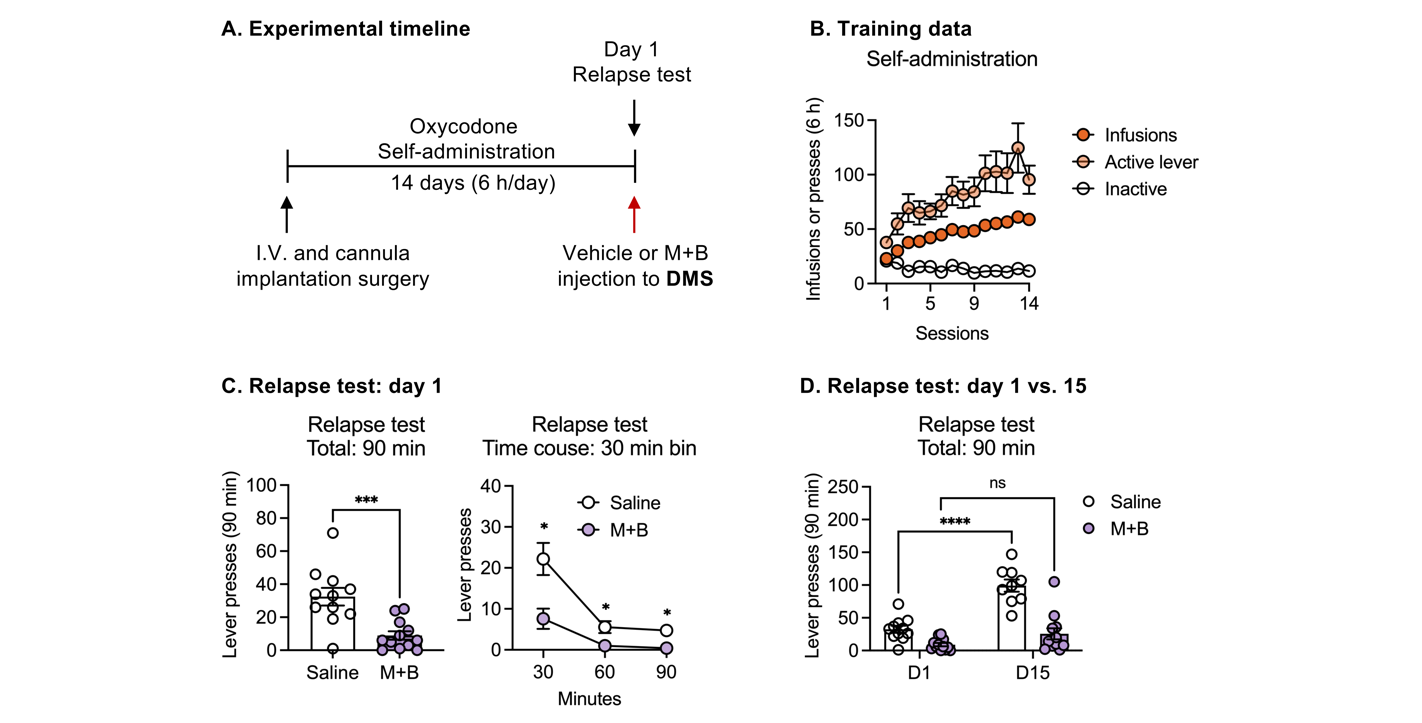
**

**Supplementary Figure 3: Effect of muscimol-baclofen (M+B) inactivation of DMS on oxycodone seeking during early abstinence.** (**A**) Experimental timeline. (**B**) Oxycodone self-administration training. Data show mean ± SEM of number of infusions and active and inactive lever presses during the training phase. (**C**) Relapse test on day 1. Data shows mean ± SEM number of active lever presses during the 90-min test session (left) or 30-min bin timecourse (right) after saline or muscimol-baclofen (50 + 50 ng per side) injection into DMS. n = 11 to 12 rats per dose. (**D**) Day 1 vs. day 15 relapse test. Data show mean ± SEM number of active lever presses during the 90-min test sessions. DMS: dorsomedial striatum. M+B: muscimol-baclofen. D1: day 1. D15: day 15. ****p<0.0001, ***p<0.001, *p<0.05.

**
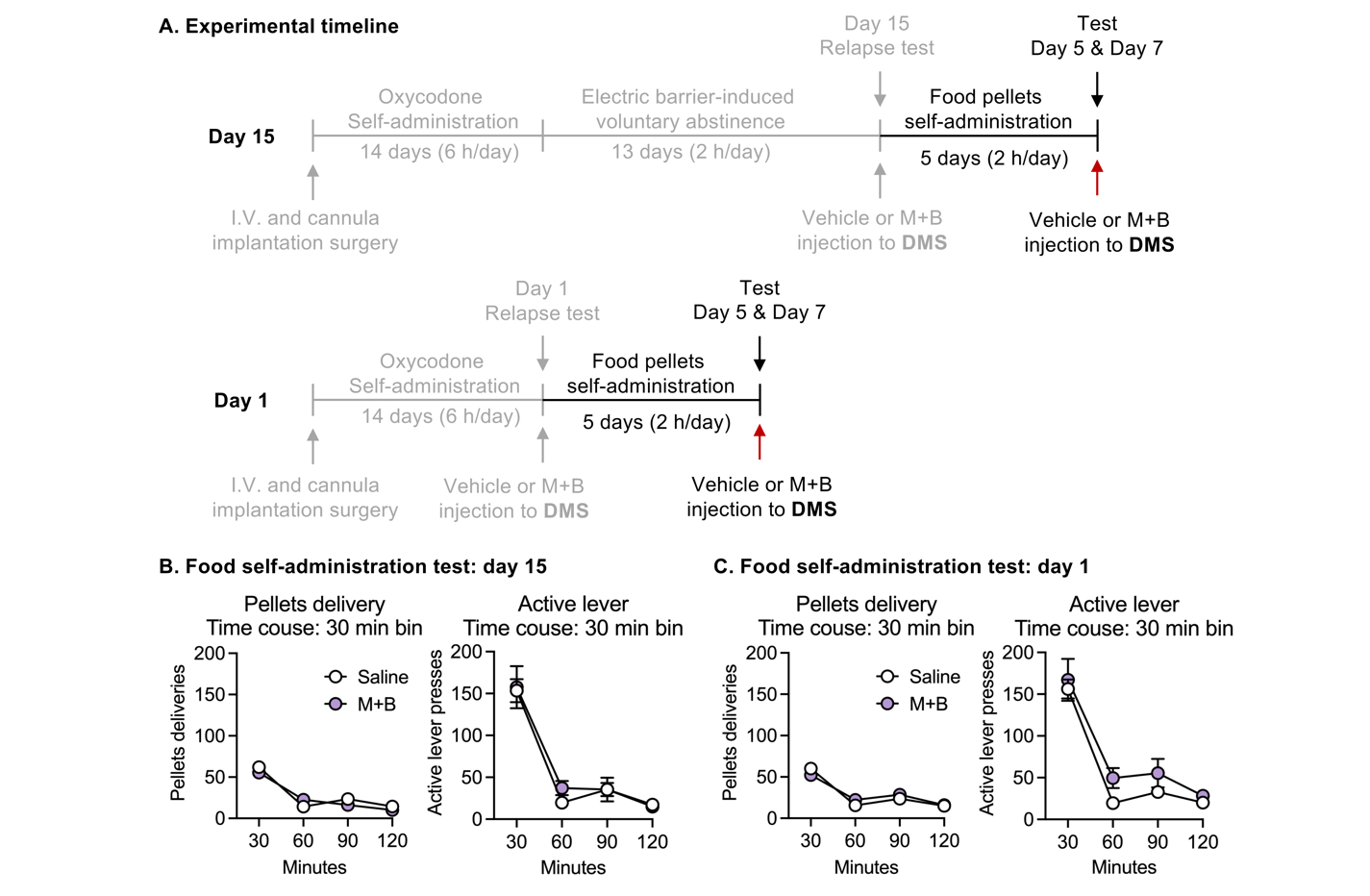
**

**Supplementary Figure 4: Effect of muscimol-baclofen (M+B) inactivation of DMS on food self-administration.** (A) Experimental timeline. (B) Food pellets self-administration test. Data show means ± SEM of number of pellet deliveries (2 pellets per delivery) active lever presses during the 120-min test sessions after saline or muscimol-baclofen (50 + 50 ng per side) injections (counterbalanced) into DMS. n = 19, within-subjects testing. DMS: dorsomedial striatum. M+B: muscimol-baclofen.

**References**

1 Caprioli D, Venniro M, Zeric T, Li X, Adhikary S, Madangopal R, et al. Effect of the novel positive allosteric modulator of mGluR2 AZD8529 on incubation of methamphetamine craving after prolonged voluntary abstinence in a rat model. Biological psychiatry. 2015;78(7):463.

2 Caprioli D, Venniro M, Zhang M, Bossert JM, Warren BL, Hope BT, Shaham Y. Role of dorsomedial striatum neuronal ensembles in incubation of methamphetamine craving after voluntary abstinence. Journal of Neuroscience. 2017;37(4):1014-27.

3 Venniro M, Zhang M, Caprioli D, Hoots JK, Golden SA, Heins C, et al. Volitional social interaction prevents drug addiction in rat models. Nature neuroscience. 2018;21(11):1520-29.

4 Cooper A, Barnea-Ygael N, Levy D, Shaham Y, Zangen A. A conflict rat model of cue-induced relapse to cocaine seeking. Psychopharmacology. 2007;194:117-25.

5 Fredriksson I, Applebey SV, Minier-Toribio A, Shekara A, Bossert JM, Shaham Y. Effect of the dopamine stabilizer (-)-OSU6162 on potentiated incubation of opioid craving after electric barrier-induced voluntary abstinence. Neuropsychopharmacology. 2020;45(5):770-79.

6 Lu H, Zou Q, Gu H, Raichle ME, Stein EA, Yang Y. Rat brains also have a default mode network. Proceedings of the National Academy of Sciences. 2012;109(10):3979-84.

7 Fredriksson I, Tsai P-J, Shekara A, Duan Y, Applebey SV, Lu H, et al. Orbitofrontal cortex and dorsal striatum functional connectivity predicts incubation of opioid craving after voluntary abstinence. Proceedings of the National Academy of Sciences. 2021;118(43):e2106624118.

8 Lu H, Patel S, Luo F, Li SJ, Hillard CJ, Ward BD, Hyde JS. Spatial correlations of laminar BOLD and CBV responses to rat whisker stimulation with neuronal activity localized by Fos expression. Magnetic Resonance in Medicine: An Official Journal of the International Society for Magnetic Resonance in Medicine. 2004;52(5):1060-68.

9 Mandeville JB, Marota JJ, Kosofsky BE, Keltner JR, Weissleder R, Rosen BR, Weisskoff RM. Dynamic functional imaging of relative cerebral blood volume during rat forepaw stimulation. Magnetic resonance in medicine. 1998;39(4):615-24.

10 Cox RW. AFNI: software for analysis and visualization of functional magnetic resonance neuroimages. Computers and Biomedical research. 1996;29(3):162-73.

11 Avants BB, Tustison NJ, Song G, Cook PA, Klein A, Gee JC. A reproducible evaluation of ANTs similarity metric performance in brain image registration. Neuroimage. 2011;54(3):2033-44.

12 Smith SM, Jenkinson M, Woolrich MW, Beckmann CF, Behrens TE, Johansen-Berg H, et al. Advances in functional and structural MR image analysis and implementation as FSL. Neuroimage. 2004;23:S208-S19.

13 Duan Y, Tsai P-J, Salmeron BJ, Hu Y, Gu H, Lu H, et al. Compulsive drug-taking is associated with habenula–frontal cortex connectivity. Proceedings of the National Academy of Sciences. 2022;119(50):e2208867119.
